# Quantitative assessment of atherosclerotic plaques on ^18^F-FDG PET/MRI: comparison with a PET/CT hybrid system

**DOI:** 10.1007/s00259-016-3308-6

**Published:** 2016-01-27

**Authors:** Xiang Li, Daniel Heber, Ivo Rausch, Dietrich Beitzke, Marius E. Mayerhoefer, Sazan Rasul, Michael Kreissl, Markus Mitthauser, Wolfgang Wadsak, Markus Hartenbach, Alexander Haug, Xiaoli Zhang, Christian Loewe, Thomas Beyer, Marcus Hacker

**Affiliations:** Division of Nuclear Medicine, Department of Biomedical Imaging and Image-guided Therapy, Medical University of Vienna, Währinger Gürtel 18-20, 1090 Vienna, Austria; Division of Cardiovascular and Interventional Radiology, Department of Biomedical Imaging and Image-guided Therapy, Medical University of Vienna, Vienna, Austria; Center for Medical Physics and Biomedical Engineering, General Hospital Vienna, Medical University of Vienna, Vienna, Austria; Division of General and Pediatric Radiology, Department of Biomedical Imaging and Image-guided Therapy, Medical University of Vienna, Vienna, Austria; Department of Nuclear Medicine, Klinikum Augsburg, Augsburg, Germany; Department of Nuclear Medicine, State Key Laboratory of Cardiovascular Disease, Fuwai Hospital, National Center for Cardiovascular Diseases, Beijing, China

**Keywords:** Atherosclerosis, Carotid plaque, Inflammation, ^18^F-FDG, PET/CT, PET/MRI

## Abstract

**Purpose:**

PET with ^18^F-FDG has the potential to assess vascular macrophage metabolism. ^18^F-FDG is most often used in combination with contrast-enhanced CT to localize increased metabolism to specific arterial lesions. Novel ^18^F-FDG PET/MRI hybrid imaging shows high potential for the combined evaluation of atherosclerotic plaques, due to the superior morphological conspicuity of plaque lesions. The purpose of this study was to evaluate the reliability and accuracy of ^18^F-FDG PET/MRI uptake quantification compared to PET/CT as a reference standard in patients with carotid atherosclerotic plaques.

**Methods:**

The study group comprised 34 consecutive oncological patients with carotid plaques who underwent both PET/CT and PET/MRI with ^18^F-FDG on the same day. The presence of atherosclerotic plaques was confirmed by 3 T MRI scans. Maximum standardized uptake values (SUV_max_) for carotid plaque lesions and the average SUV of the blood pool within the adjacent internal jugular vein were determined and target-to-blood ratios (TBRs, plaque to blood pool) were calculated.

**Results:**

Atherosclerotic lesions with maximum colocalized focal FDG uptake were assessed in each patient. SUV_max_ values of carotid plaque lesions were significantly lower on PET/MRI than on PET/CT (2.3 ± 0.6 vs. 3.1 ± 0.6; *P* < 0.01), but were significantly correlated between PET/CT and PET/MRI (Spearman’s *r* = 0.67, *P* < 0.01). In contrast, TBR_max_ values of plaque lesions were similar on PET/MRI and on PET/CT (2.2 ± 0.3 vs. 2.2 ± 0.3; *P* = 0.4), and again were significantly correlated between PET/MRI and PET/CT (Spearman’s *r* = 0.73, *P* < 0.01). Considering the increasing trend in SUV_max_ and TBR_max_ values from early to delayed imaging time-points on PET/CT and PET/MRI, respectively, with continuous clearance of radioactivity from the blood, a slight underestimation of TBR_max_ values may also be expected with PET/MRI compared with PET/CT.

**Conclusion:**

SUV_max_ and TBR_max_ values are widely accepted reference parameters for estimation of the radioactivity of atherosclerotic plaques on PET/CT. However, due to a systematic underestimation of SUV_max_ and TBR_max_ with PET/MRI, the optimal cut-off values indicating the presence of inflamed plaque tissue need to be newly defined for PET/MRI.

## Introduction

An excessively high rate of sudden death due to cerebrovascular diseases in apparently healthy individuals without prior symptoms has been reported [1]. The most frequent cause is a sudden rupture of an unstable atherosclerotic plaque, which results in either thrombotic occlusion at the site of rupture or distal embolization [2, 3]. It remains a major challenge for preventative medicine to identify high-risk patients who would benefit from intervention prior to a rupture of a nascent plaque. In the evaluation of atherosclerotic plaques, the thin cap fibroatheroma is recognized as the plaque type with the highest risk of rupture [4]. On conventional imaging, MRI, because of its excellent soft tissue contrast, provides the unique potential to identify most of the pathomorphological key features of vulnerable carotid plaques.

One of the primary determinants of atherosclerotic plaque ruptures is inflammation, which leads to a high number of strokes and myocardial infarctions [5]. PET is a method for investigating the pathophysiology and propagation of diseases with the help of radiotracers [6, 7]. Numerous metabolic and pathophysiological biomarkers associated with plaque vulnerability have been investigated as targets for PET imaging. Among them, ^18^F-FDG is the PET tracer that is most commonly used to assess inflamed plaques by evaluating the corresponding glucose metabolism [8–11], particularly of resident macrophages, which most avidly accumulate ^18^F-FDG. However, PET imaging is limited by a relatively low spatial resolution, and thus the use of morphological imaging is needed to localize the tracer uptake [12], which can be provided by dual modality imaging systems such as PET/CT and PET/MRI. PET/CT allows fast data acquisition and the CT dataset can be reliably used as a transmission scan for attenuation correction (AC) of the PET data [13]. It has also been shown that vessel calcifications on non-contrast CT scans correlate significantly with the patient’s overall plaque burden [14].

With regard to characterization of arterial plaques, MRI possesses several inherent advantages over CT, including higher spatial resolution, an excellent soft tissue contrast, and a lack of ionizing radiation. Furthermore, in the setting of PET/MRI hybrid imaging, PET and MRI can be performed simultaneously, which enables real-time MRI motion and partial-volume correction of the PET data. Therefore, the combination of 3-T MRI with simultaneous PET imaging for comprehensive evaluation of carotid plaques has great potential [15], but with the caveat of suboptimal PET AC based on MRI (MR-AC) [16]. The MR signal intensity is not proportional to the amount of gamma photon attenuation [17, 18], so that MR-AC is more challenging than CT-AC. Previous research has demonstrated a significant correlation of the tracer uptake ratio from PET using MR-AC with the ratio from PET using CT-AC in head and neck cancer [19]. Nevertheless, reliable quantification of vessel wall inflammation by PET/MRI has not yet been demonstrated. The maximum standardized uptake value (SUV_max_) is the most commonly used value for quantification of radioactivity on PET. In addition, the target-to-blood pool ratio (TBR) is widely accepted for measuring the inflammatory activity of atherosclerotic plaques on PET [11].

In the present study of patients with carotid atherosclerotic plaques, we examined the accuracy and reliability of clinical PET/MR imaging, based on the SUV and TBR values in comparison to PET/CT as reference.

## Materials and methods

### Patients

We reviewed PET/CT and PET/MR imaging studies in 247 oncological patients who underwent both procedures for staging and restaging of different cancer types between March 2014 and March 2015. Of these patients, 34 showed colocalized focal uptake (TBR ≥1.6) at the carotid bifurcation on both PET/CT and PET/MRI, and were included in this study. Baseline data for the study patients, including type of cancer, age, gender, BMI, blood sugar concentration and diabetes, are presented in Table 1. This retrospective image analysis was approved by the local ethics committee. For identification of patients with carotid plaque lesions, MR image sets from PET/MRI were used.

**Table 1:** Baseline characteristics of the study population

Characteristic	Value
No. of patients	34
Age (years), mean ± SD (range)	61 ± 9 (52 – 84)
Male, *n* (%)	23 (64)
Body mass index (kg/m^2^), mean ± SD (range)	26 ± 3.9 (19 – 31)
Plasma glucose (mg/dL), mean ± SD (range)	104 ± 15 (71 – 128)
Diabetes, *n* (%)	3 (8)
Cancer type, *n* (%)	
Lymphoma	27 (79)
Head and neck cancer	4 (12)
Thyroid cancer	3 (9)

### PET/CT imaging

All patients underwent an ^18^F-FDG PET/CT scan on a dedicated PET/CT system (Siemens Biograph TPTV 64; Siemens, Knoxville, TN) consisting of an LSO-based full-ring PET scanner and a 64-row multidetector CT scanner. In 30 patients (88 %) contrast medium was injected for the CT scan. On the day of the scan, patients fasted for at least 6 h; their mean glucose level was 103 ± 14.4 mg/dL (range 71 – 128 mg/dL). ^18^F-FDG was injected intravenously at 4.2 ± 0.9 MBq/kg body weight (range 3.3 – 5.5 MBq/kg). After an uptake period of 74 ± 13 min (range 47 – 92 min), transmission data were acquired from the base of the skull or the vertex to the proximal thighs. PET emission data were then acquired in 3D mode with a 168 × 168 matrix (pixel size 4.2 mm) with an emission time of 3 min per bed position. After decay and scatter correction, PET data were reconstructed iteratively, applying point-spread function (PSF) correction (TrueX algorithm) using four iterations and 21 subsets. The CT data were used for AC.

### PET/MR imaging

After PET/CT, ^18^F-FDG PET/MR imaging was performed on a Biograph mMR system (Siemens Healthcare, Erlangen, Germany) incorporating a 3-T MRI scanner. The PET/MRI acquisition was started 140 ± 21 min (range 100 – 189 min) after administration of ^18^F-FDG. PET/MR images were acquired in four or five bed positions with 5 min per bed position. The MR imaging component was performed with an integrated radiofrequency coil and a multistation protocol, with a slice thickness of 2 mm. AC was performed using the implemented standard four-compartment model attenuation map calculated from a Dixon-based VIBE (volumetric interpolated breath-hold examination) sequence. A 3D ordinary Poisson ordered subsets expectation maximization (OP-OSEM) algorithm with PSF correction with three iterations and 21 subsets was used for reconstruction. The image matrix size was 172 × 172 (pixel size 4.2 mm). The images were smoothed with a 3-mm full-width at half-maximum (FWHM) gaussian filter.

### Image analysis

Automatic image orientation, 3D image fusion, and image analysis for both PET/MRI and PET/CT were performed with commercially available software (Hermes Hybrid 3D; Hermes Medical Solutions, Stockholm, Sweden). In a first step, two cardiovascular radiology specialists blinded to the patients’ clinical information visually evaluated the MR images (T1-weighted VIBE Dixon sequence) for the presence of plaques at the left and right carotid artery bifurcation. In a second step, two nuclear medicine physicians blinded to the clinical information determined colocalized focal uptake of ^18^F-FDG on both PET/CT and PET/MRI for the visualized carotid plaque lesions. To assess the reproducibility of the SUVs obtained, all lesions were reassessed by the same physicians after 3 weeks. In a third step, SUV_max_ values of carotid plaques were obtained for both PET/CT and PET/MRI using a region-of-interest (ROI) approach. To calculate TBRs, respective SUV_max_ values were corrected for background blood-pool activity, which was calculated as the mean SUV of four ROIs within the lumen of both (left and right) internal jugular veins and both (left and right) external jugular veins. Carotid plaques detectable on both sides were detected in 12 patients. In those patients, contralateral ROI analysis was performed for the less prominent atherosclerotic bifurcation region. Correction for radioactive decay was applied automatically by the system for both PET/CT and PET/MRI based on the injection time. The circulation time of ^18^F-FDG is a major factor that can influence the SUV and TBR values within plaques. We performed a regression analysis to evaluate the relationship between the plaque and blood pool uptake ratios.

### Statistical methods

SPSS version 22.0 (IBM, Armonk, NY) was used for statistical analysis. Continuous variables with a normal distribution were recorded as means ± standard deviation. Spearman’s correlation coefficient was used to assess the association between PET/CT and PET/MRI SUVs and TBR values. Intraclass correlation coefficients (ICCs) with 95 % confidence intervals were calculated to test interobserver and intraobserver agreement for TBR. Two-way random ICCs greater than 0.8 were accepted as indicating excellent reproducibility. Group comparisons were made using one-way ANOVA. *P* values less than 0.05 were considered statistically significant. Regression analyses for the SUV_max_ and TBR_max_ of carotid plaques on PET/CT and PET/MRI with circulation time were performed. Bland Altman analysis was used to assess agreement between the two measurements. Data are presented as means ± standard deviations with range.

## Results

### Detection of carotid plaques

**Fig. 1:**
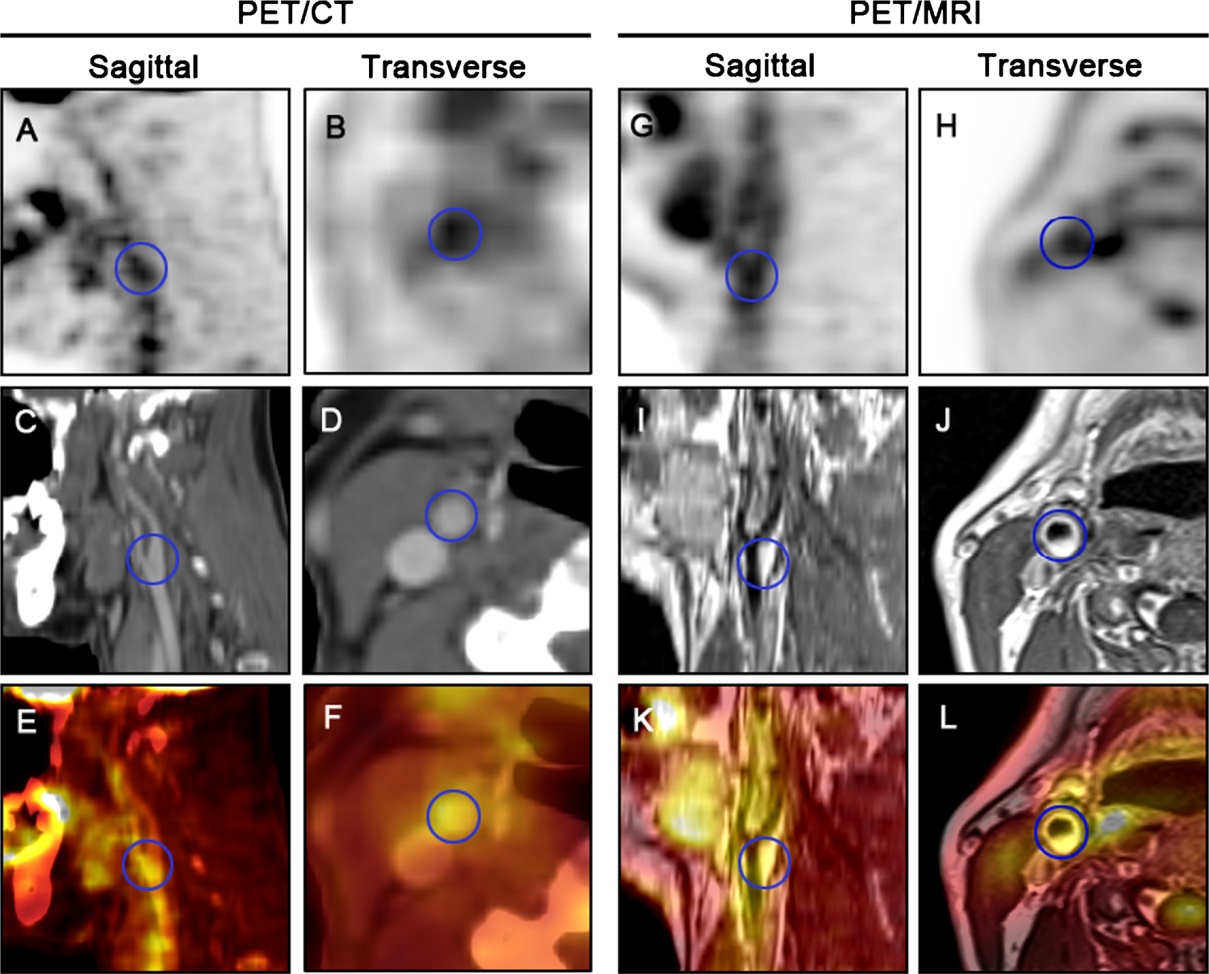
Fused PET/CT (**a**–**f**) and PET/MRI (**g**–**l**) images of the right carotid artery in a 52-year-old patient with a head and neck tumour (*blue circles* ROIs of a plaque at the carotid bifurcation). The right carotid artery shows focal pathological ^18^F-FDG uptake on the PET images with CT-based attenuation correction. Colocalized focal uptake of ^18^F-FDG is seen on the PET image with MR attenuation correction

Carotid plaques were detected in 52 patients from the MR images with T1-weighted turbo spin-echo (TSE) sequences in the neck and head region. Of these patients, 34 with colocalized focal uptake (TBR ≥1.6) on both PET/CT and PET/MRI were included in this study. The carotid plaque lesion in each of these 34 patients with the highest focal uptake was assessed on PET/CT and PET/MRI. Calcified spots (≥200 HU) were detected on carotid plaques in five patients using CT. Representative images are shown in Fig. 1.

### Quantitative analysis of PET data from PET/CT and PET/MRI

The mean ^18^F-FDG SUV_max_ of carotid plaques on PET/MRI was significantly lower than the value on PET/CT, but the values on PET/MRI and PET/CT were significantly correlated (Spearman’s *r* = 0.67, *P* < 0.01; Table 2). Blood-pool uptake was higher on PET/CT (1.4 ± 0.3, range 0.9 – 2.0) than on PET/MRI (1.1 ± 0.2, range 0.8 – 1.7; *P* = 0.1) due to clearance of ^18^F-FDG from the blood over time.

**Table 2:** Comparison of mean SUV_max_ values from PET/CT and PET/MRI for the 34 carotid plaque lesions

	SUV_max_		Spearman’s *r*	*P* value^a^
	Mean ± SD	Range		
PET/CT	3.1 ± 0.6	2.2 – 4.7	0.67	0.01
PET/MRI	2.3 ± 0.6	1.7 – 4.5		

^a^One-way ANOVA

**Fig. 2:**
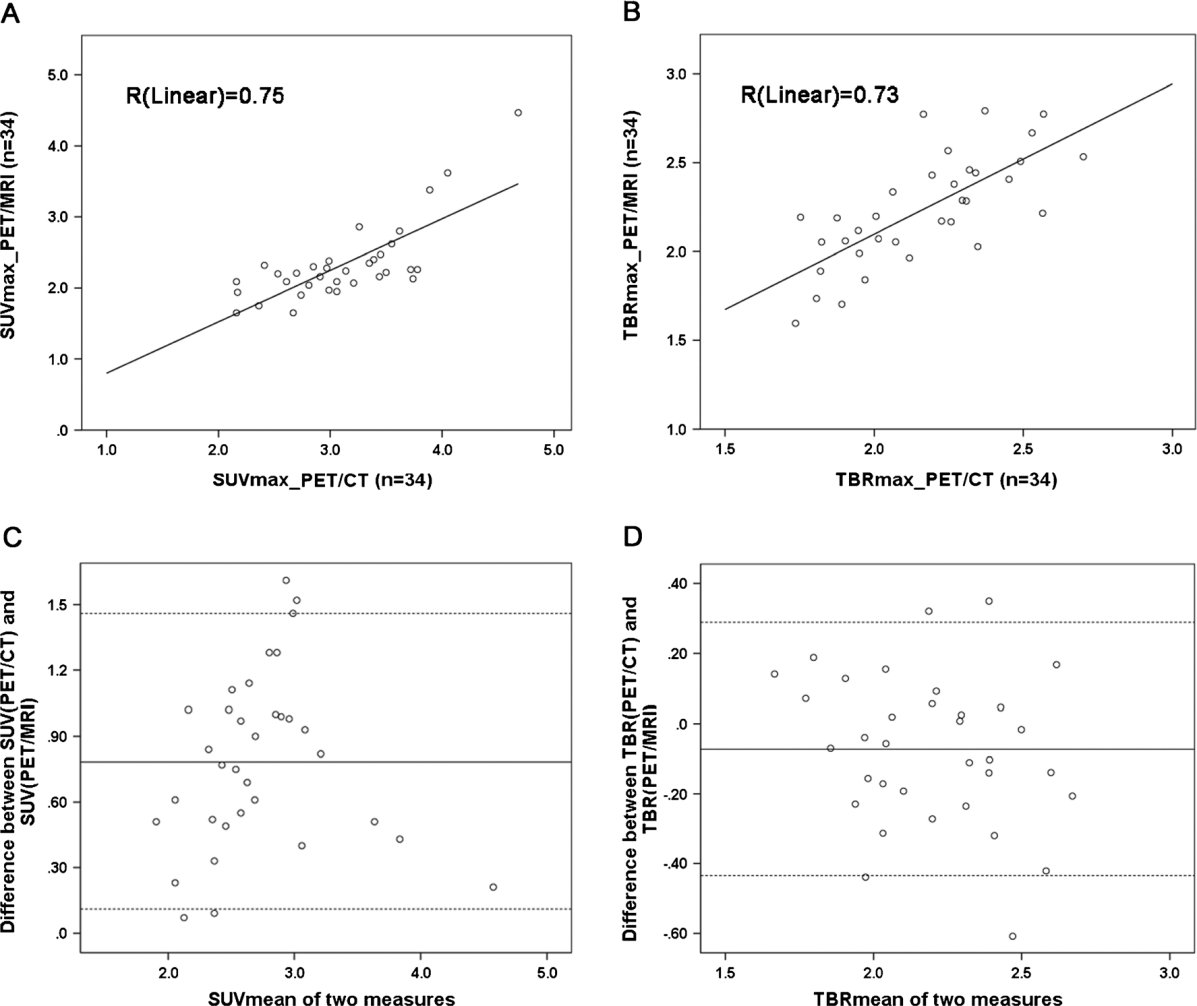
**a**, **b** Linear regression analysis of SUV (**a**) and TBR (**b**) for 34 carotid plaques obtained on ^18^F-FDG PET/MRI and ^18^F-FDG PET/CT. **c**, **d** Bland Altman analysis of the agreement between the two systems (SUV and TBR on PET/CT minus SUV and TBR on PET/MRI, with a lower bias of −0.07 for TBR_mean_ compared with a bias of 0.8 for SUV_mean_)

**Fig. 3:**
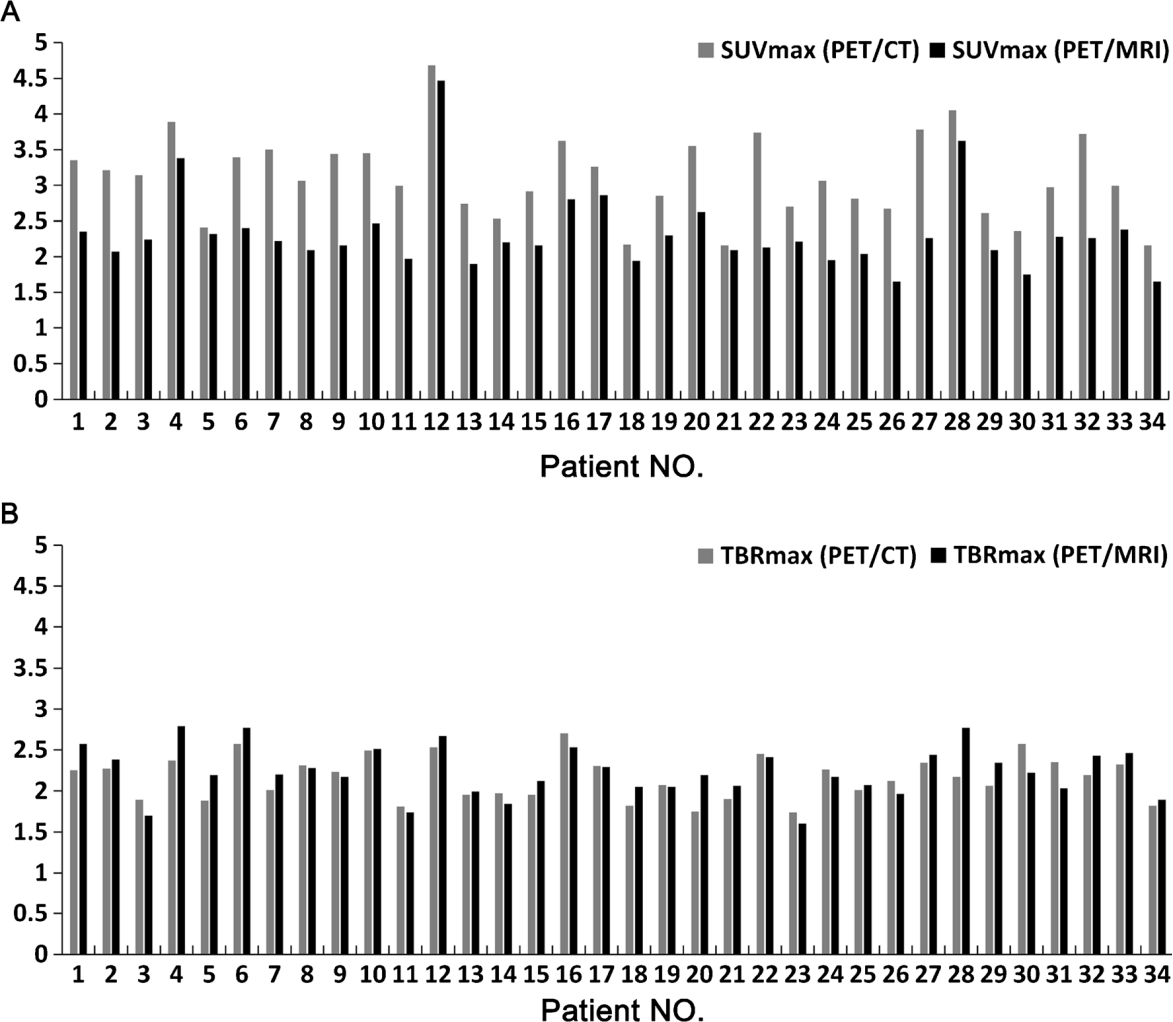
Mean SUV_max_ (**a**) and TBR_max_ (**b**) in each individual patient (*n* = 34) on ^18^F-FDG PET/CT and PET/MRI. In all patients, SUV_max_ values were higher on PET/CT, and in a majority of patients (*n* = 20), TBR_max_ values were higher on PET/MR

In contrast, there was no significant difference between the mean TBR_max_ values of plaque lesions on PET/CT and PET/MRI, and the correlation of TBR_max_ values between PET/CT and PET/MRI was even better (Spearman’s *r* = 0.73, *P* < 0.01; Table 3). The linear regression correlation coefficients (*R*) for the uptake values obtained from the two systems were 0.75 for SUV_max_ and 0.73 for TBR_max_ (Fig. 2a, b). Bland Altman analysis was used to assess agreement between the two measurements, with a lower bias for TBR_mean_ (−0.07) than the bias for SUV_mean_ (0.8) between PET/CT and PET/MRI, corresponding to a zero difference (Fig. 2c, d). In the assessment of individual patients, the SUV_max_ values from the PET/CT images were higher than those from the PET/MR images in all patients (Fig. 3a). However, in 20 patients, the TBR_max_ value from the PET/MR images was higher than that from the PET/CT images (Fig. 3b).

**Table 3:** Comparison between TBR_max_ values from PET/CT and PET/MRI for the 34 carotid plaque lesions

	TBR_max_		Spearman’s *r*	*P* value^a^
	Mean ± SD	Range		
PET/CT	2.2 ± 0.3	1.7 – 2.7	0.73	0.3
PET/MRI	2.2 ± 0.3	1.6 – 2.8		

^a^One-way ANOVA

 A comprehensive group analysis comparing patients with TBR(PET/CT) < TBR(PET/MRI) (group 1, *n* = 20) and patients with TBR(PET/CT) ≥ TBR(PET/MRI) (group 2, *n* = 14) was performed (Table 4), which demonstrated a lower blood-pool activity in group 1 with a longer average circulation time on PET/MR scans, and patients in group 1 had slightly higher mean SUV_max_ and TBR_max_ on PET/MR scans. In 12 patients, a significantly higher uptake was detected on the plaque side than on the less prominent side on both PET/CT and PET/MRI; likewise, significantly higher TBR values were obtained for plaque lesions than for less prominent lesions on both PET/CT and PET/MRI (Table 5).

**Table 4:** Group analysis comparing patients with TBR(PET/CT) < TBR(PET/MRI) (group 1, *n* = 20) and patients with TBR(PET/CT) ≥ TBR(PET/MRI) (group 2, *n* = 14)

Parameter	Group 1		Group 2		*P* value^a^
	Mean ± SD	Range	Mean ± SD	Range	
SUV_max_					
PET/CT	3.1 ± 0.7	2.2 – 4.7	3.0 ± 0.4	2.5 – 3.7	0.5
PET/MRI	2.4 ± 0.7	1.7 – 4.5	2.2 ± 0.3	1.7 – 2.9	0.2
Blood pool					
PET/CT	1.4 ± 0.3	1.1 – 2.0	1.4 ± 0.2	0.9 – 1.7	0.3
PET/MRI	1.0 ± 0.2	0.9 – 1.7	1.1 ± 0.2	0.8 – 1.4	0.6
TBR_max_					
PET/CT	2.1 ± 0.3	1.8 – 2.6	2.2 ± 0.3	1.7 – 2.7	0.5
PET/MRI	2.3 ± 0.3	1.9 – 2.8	2.1 ± 0.3	1.6 – 2.5	0.08
Circulation time (min)					
PET/CT	74.6 ± 11.8	47 – 92	73.2 ± 11.6	55 – 92	0.7
PET/MRI	141.5 ± 19.8	101 – 173	132.7 ± 23.5	100 – 189	0.4

^a^One-way ANOVA

**Table 5:** SUV_max_ and TBR_max_ values from contralateral lesions (*n* = 12) and carotid plaque lesions (*n* = 12) as obtained on ^18^F-FDG PET/CT and ^18^F-FDG PET/MRI

	PET/CT						PET/MRI			
	Contralateral side			Plaque		*P* value^a^	Contralateral side	Plaque	*P* value^a^	
	Mean ± SD	Range	Mean ± SD	Range	Mean ± SD		Range	Mean ± SD		Range
SUV	2.5 ± 0.4	1.8 – 3.2	2.4 – 4.5)	2.4 – 4.5	<0.01	1.7 ± 0.3	1.2 – 2.3	2.3 ± 0.5	1.7 – 3.7	<0.01
TBR	1.7 ± 0.2	1.2 – 2.1	2.2 ± 0.3	1.7 – 2.7	<0.01	1.6 ± 0.3	1.1 – 1.9	2.2 ± 0.3	1.6 – 2.8	<0.01

^a^One-way ANOVA

**Fig. 4:**
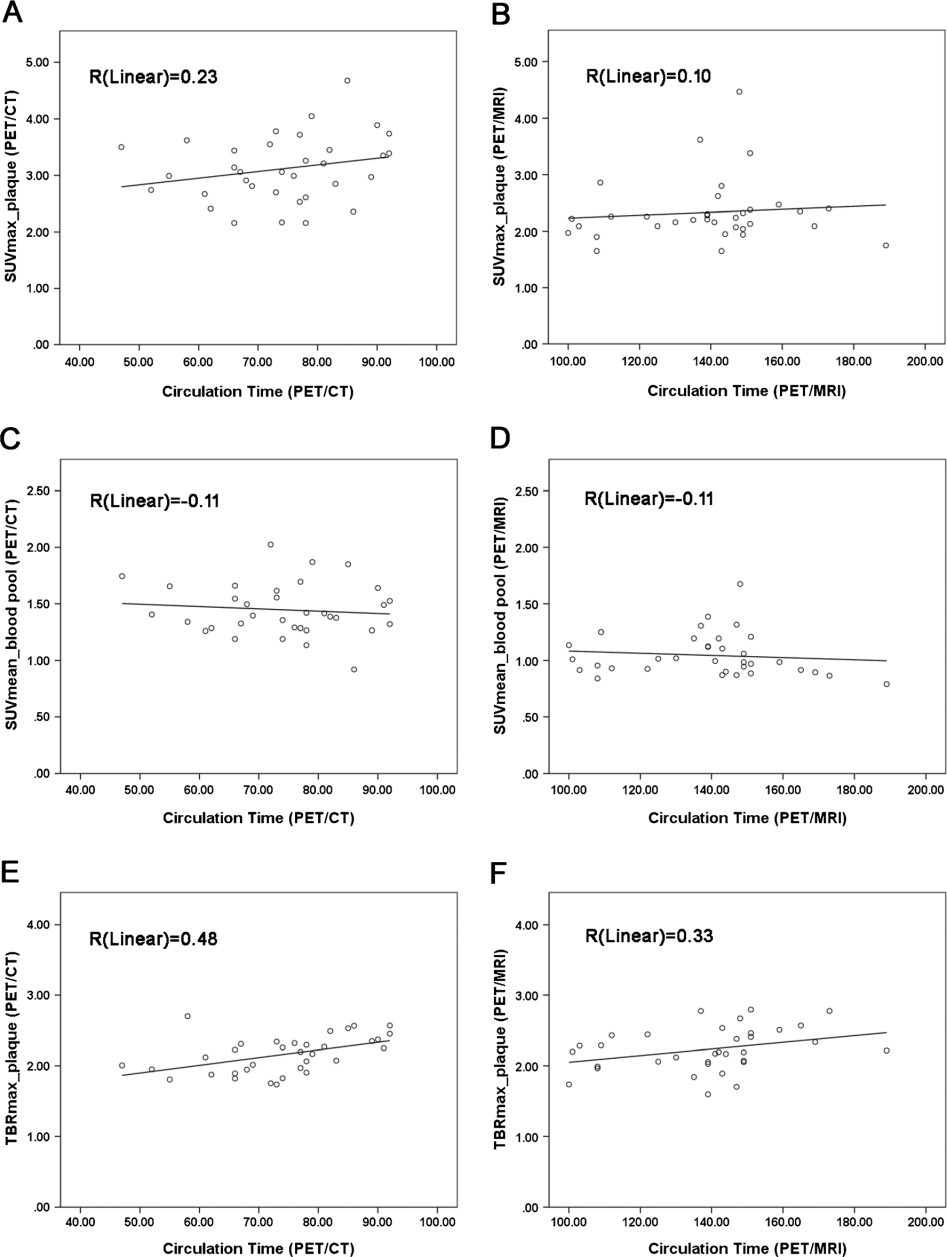
Regression analysis between amount of radioactivity and circulation time (minutes). Both SUV_max_ and TBR_max_ of carotid plaque, on both PET/CT and PET/MRI, showed an upward trend from early to late imaging time-points with continued decreasing activity as a result of blood clearance

In the regression analyses between uptake value and circulation time on PET/CT and PET/MRI, both SUV_max_ and TBR_max_ in the plaque showed an up-trend from the early time to the later time on the PET/CT and PET/MRI, respectively, and continued clearance of blood was observed as well (Fig. 4).

### Intraclass correlation coefficients

There were excellent interobserver (*R* = 0.87) and intraobserver (*R* = 0.96) correlations, along with 95 % confidence intervals for ^18^F-FDG SUVs.

## Discussion

Among cardiovascular imaging modalities, MRI has emerged as a powerful tool for the assessment of the composition of plaques, while PET has been investigated widely in cardiovascular disease because it offers functional quantitative information at the molecular level [20, 21]. In the present study, we compared the quantification of glucose uptake in carotid plaque lesions on PET/MRI, with PET/CT as the standard of reference. The main result was that quantification of FDG uptake in the carotid arteries is feasible with integrated PET/MRI, and TBR can be measured with similar accuracy to that obtained with PET/CT. Qualitatively, this had already been shown by one case study of simultaneous PET/MR imaging and MR angiography of atherosclerotic plaque in the carotid artery, which revealed significant ^18^F-FDG uptake in carotid plaques [22]. Nevertheless, SUV_max_ values from PET/MR were significantly lower than those from PET/CT, which is in line with the results of previous studies comparing quantitative values from PET/MR with those from PET/CT in oncological imaging. Underestimation of SUV_max_ on PET/MR has been observed for different organs (liver, lung, spleen, bone and muscle) [23].

Furthermore, in an initial feasibility study on the quantification of vessel wall signals in carotid arteries of HIV patients at increased risk of atherosclerosis, Ripa et al. found significantly lower ^18^F-FDG SUV_mean_ and SUV_max_ values on PET/MRI than on PET/CT [24]. This difference in SUV may have been for physiological and/or technical reasons. SUV is a snapshot of the accumulated FDG in a certain region. As FDG uptake is a dynamic process, SUV can change with time after tracer injection [25]. Nevertheless, a previous study of ^18^F-FDG kinetics in atherosclerotic patients showed no significant differences in SUV_max_ and TBR_max_ values between 1 and 3 h after tracer injection [26]. Furthermore, in our study, there was no significant difference in TBR values during the same period. Therefore, a substantial physiological change in SUV between 74 ± 13 and 140 ± 21 min after injection would not be expected to cause the great differences in SUV found in the present study. Blomberg et al. also demonstrated significantly increased TBR on delayed ^18^FDG PET/CT (180 min) compared with early imaging (90 min) due to the declining blood pool and the extended uptake of tracer [27]. However, in our study, we did not find significantly increased TBR values on PET/MRI compared with PET/CT. To summarize, in our study, there was a lower mean SUV_max_ and no significant increase in mean TBR_max_ values on later PET/MRI scans compared with earlier PET/CT scans, which indicated systematic underestimations of both SUV and TBR in the evaluation of plaque uptake. As shown in Table 4, patients with higher TBR_max_ values on PET/MRI than on PET/CT showed lower blood-pool activities and longer circulation times, on average. This corresponds to previous findings [27].

Analysis of agreement between PET/CT and PET/MRI measurements showed similar regression ratios for SUV_max_ and TBR_max_ values between the two systems, whereas Bland-Altman analysis demonstrated a lower bias of −0.07 for mean TBR compared with 0.8 for SUV, which indicates a higher agreement between PET/CT and PET/MRI measurement for TBR (Fig. 2).

Technical aspects affecting the SUV include differences in the reconstruction method, postfiltering, partial volume effects and differences in attenuation and scatter correction. The differences in reconstruction in this study were minimal. Both PET/CT and PET/MR images were reconstructed using an OSEM algorithm with PSF correction developed by the same vendor. The most prominent difference was the use of different numbers of iterations for the convergence, and therefore the recovery of activity was dependent on the number of iterations, and, as shown by Hudson and Larkin [28], on the product of iterations and subsets. A study by Knäusl et al. [29] showed that between iteration–subset products of 84 and 64 there are differences of 1 – 9 % in the SUVmax for spheres (11.5 – 0.3 ml in size) in a modified NEMA image quality phantom. We used the same reconstruction algorithm in our study for the PET/CT examinations. With regard to the differences in postfiltering, a study by Tong et al. [30] found a reduced contrast recovery of between 5 % and 8 % for spheres of 10 – 22 mm in diameter for a similar reconstruction, after applying a 4-mm FWHM gaussian filter.

 Taking these findings into account, the underestimation of SUV can be partially (we estimate around 10 % of the mean of 25 %) explained by the differences in the reconstruction methods used. The partial volume effect was not an issue in the current study, as it is dependent on pixel size, and similar pixel sizes were used for PET/CT and PET/MRI reconstructions. The main cause of the underestimation is thought to have resulted from differences in AC. As bone is ignored on standard MR-AC [17], and the proportion of bone is rather high in the neck region, the omission of bone from AC could cause a substantial under-correction of the activity values, and thus of the SUV [31]. A solution to the issue of inaccurate AC in this case could be to use TBR rather than SUV. If the reference region used to normalize lesion uptake is located close to the lesion itself, similar underestimation can be expected for both values, and therefore the underestimations will cancel each other out, which is supported by the findings of the present study. Moreover, SUV quantification in vessels is affected by adjacent blood-pool activity. There is thus a widely accepted consensus that TBR, which is corrected for the interference of blood-pool activity, should be used instead of SUV. For the calculation of TBR, we used the mean blood activity from both internal jugular veins and external jugular veins as the blood pool activity. To avoid registration problems, we chose the carotid bifurcation region for the present study, because this region is particularly prone to plaque formation [32], and enables high registration accuracy when estimating the colocalized uptake of ^18^F-FDG on PET/CT and PET/MRI.

Considering the reduced radiation dose and superior lesion discernment, PET/MRI might be used as an alternative to PET/CT in the evaluation of carotid plaque lesions. However, AC in PET/MRI is still challenging, and underestimations of both SUV and TBR were observed in this study; thus, a reliable quantitative criterion based on cut-off uptake ratios for inflamed plaque is required. In addition, high cost, as well as longer examination times, also limit the application of PET/MRI.

There were several limitations to this study. First, the study included a limited number of oncological patients, and therefore the findings might not be applicable to the general population. Second, advanced morphological assessment of carotid plaques was not performed. Third, histological or autoradiographic validation of ^18^F-FDG uptake quantification in the carotid plaques could not be provided. Fourth, nonrandomization of imaging sequences and distinct imaging reconstruction methods may have caused confounded uptake ratios. However, such studies have previously been published for PET/CT evaluation [8], and this is why PET/CT was chosen as the reference standard in the present study.

### Conclusion

SUV_max_ and TBR_max_ values are the widely accepted reference parameters for estimation of the radioactivity of atherosclerotic plaques on PET/CT. However, due to systematic underestimation of SUV_max_ and TBR_max_ on PET/MRI, the optimal cut-off values indicating the presence of inflamed plaque tissue need to be newly defined for PET/MRI.
